# Dissecting the structural basis of MEIG1 interaction with PACRG

**DOI:** 10.1038/srep18278

**Published:** 2016-01-04

**Authors:** Wei Li, Ninad M. Walavalkar, William A. Buchwald, Maria E. Teves, Ling Zhang, Hong Liu, Stephanie Bilinovich, Darrell L. Peterson, Jerome F. Strauss III, David C. Williams Jr, Zhibing Zhang

**Affiliations:** 1 Department of Obstetrics and Gynecology, Virginia Commonwealth University, Richmond, VA, 23298; 2 Department of Pathology and Laboratory Medicine, University of North Carolina, Chapel Hill, NC 27599.; 3 School of Public Health, Wuhan University of Science and Technology, Wuhan, Hubei, 430065, China; 4 Department of Biochemistry & Molecular Biology, Virginia Commonwealth University, Richmond, VA, 23298.

## Abstract

The product of the meiosis-expressed gene 1 (MEIG1) is found in the cell bodies of spermatocytes and recruited to the manchette, a structure unique to elongating spermatids, by Parkin co-regulated gene (PACRG). This complex is essential for targeting cargo to the manchette during sperm flagellum assembly. Here we show that MEIG1 adopts a unique fold that provides a large surface for interacting with other proteins. We mutated 12 exposed and conserved amino acids and show that four of these mutations (W50A, K57E, F66A, Y68A) dramatically reduce binding to PACRG. These four amino acids form a contiguous hydrophobic patch on one end of the protein. Furthermore, each of these four mutations diminishes the ability of MEIG1 to stabilize PACRG when expressed in bacteria. Together these studies establish the unique structure and key interaction surface of MEIG1 and provide a framework to explore how MEIG1 recruits proteins to build the sperm tail.

Mouse meiosis expressed gene 1 (*Meig*1) was originally identified during a search for mammalian genes potentially involved in meiotic processes^1^,^2^,^3^, and subsequent studies suggested that MEIG1 might play roles in meiosis in both male and female germ cells^4^,^5^,^6^,^7^. However, studies from two independent laboratories highlighted its role in spermiogenesis, the final phase of spermatogenesis in which elongating/condensing spermatids form a flagellum and differentiate into sperm. We discovered that *Meig1* mutant male mice are all infertile due to severe defects in spermiogenesis^8^. The homozygous mutant females were fertile. When the floxed *Meig1* mice were crossed to cell-type specific Cre transgenic mice, we discovered that MEIG1′s primary role is in germ cells^9^. The male infertility and spermatogenesis defects in MEIG1-deficient mice were independently replicated using another mutant mouse generated by another laboratory^10^.

MEIG1′s function in spermiogenesis has been further studied in our laboratory. Using yeast two-hybrid screen, we found that Parkin co-regulated gene (PACRG) to be a major binding partner^8^. Mice lacking MEIG1 and PACRG share an identical reproductive phenotype of male infertility due to a defect in spermiogenesis^11^,^12^,^13^. Our recent studies demonstrated that PACRG protein is expressed at approximately day 30 after birth during the first wave of spermatogenesis, when the germ cells differentiate into elongating spermatids. The protein is localized in the manchette, a structure unique to male germ cells, which is only present in the elongating spermatids where it is thought to transport cargos and direct formation of the sperm flagellum^14^,^15^. MEIG1 protein is expressed much earlier than PACRG. It is localized in the whole cell body of spermatocytes and round spermatids, but migrates to the manchette when germ cells develop into elongating spermatids. This targeting process is dependent on PACRG^16^. Thus, MEIG1 and PACRG form a complex in the manchette and which is believed to transport cargo for construction of the sperm tail.

One of the cargos needed to construct the sperm tail axoneme is SPAG16L. The mammalian *Spag16* gene is the orthologue of *Chlamydomonas reinhardtii Pf20*, which is essential for flagellar motility^8^,^9^,^10^. Using the C-terminus of SPAG16L protein as bait, a yeast two-hybrid screen identified MEIG1 as a binding partner^10^. In MEIG1 and PACRG-deficient mice, SPAG16L fails to localize to the manchette, consistent with the notion that the MEIG1/PACRG complex is involved in transporting and targeting sperm tail components to the site of flagellum assembly.

The mouse *Meig1* gene yields multiple transcripts expressed in both germ cells and somatic cells in the testis, but all these transcripts translate into the same protein^3^,^8^. MEIG1 is a small protein, with only 88 amino acids, and no known functional domains have been identified by bioinformatics analyses. How does this small protein associate with different proteins, such as PACRG and SPAG16L? To answer this question, we took advantage of nuclear magnetic resonance (NMR) to resolve the MEIG1 structure. We discovered that MEIG1 adopts a unique fold that provides a large contact surface for binding to other proteins. The shape of MEIG1 resembles a dumbbell, such that associated proteins can bind either of two opposing concave surfaces or the two convex ends of the dumbbell. Based on this structure we mutated 12 solvent exposed amino acids in MEIG1 protein that may mediate interaction with other proteins. Four amino acids, W50, K57, F66, and Y68, which are on the same large globular domain, significantly affect binding to PACRG. In addition, PACRG is not stable in bacteria, but could be stabilized by wild-type MEIG1. The ability to stabilize PACRG was also reduced in the four mutant MEIG1 proteins. Finally, a double mutation (W50A/Y68A) further reduced the binding strength and ability to stabilize PACRG as compared to the single mutations alone. These studies established the structure of MEIG1, and the amino acids that mediate interactions with PACRG. They provide a basis for exploring the structural mechanisms of MEIG1 interaction with other proteins involved in formation of the sperm tail.

## Results and Discussion

### Mouse MEIG1 adopts a unique fold with a large solvent exposed surface area

**Figure 1:**
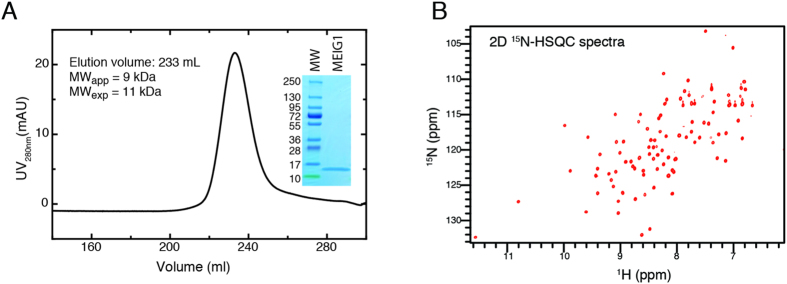
MEIG1 is monomeric in isolation. (**A**) MEIG1 protein elutes as a single peak on a Superdex 75 26/60 gel filtration column (GE Healthcare) at a volume of 233 mL, consistent with a monomer (apparent molecular weight of ~9 kDa as compared to the expected molecular weight of 11 kDa). SDS-PAGE analysis shows that the protein migrates as a single band at an appropriate molecular weight. (**B**) A 2D ^15^N-HSQC spectrum of MEIG1 shows sharp and well dispersed resonances consistent with a stable folded monomer.

**Figure 2:**
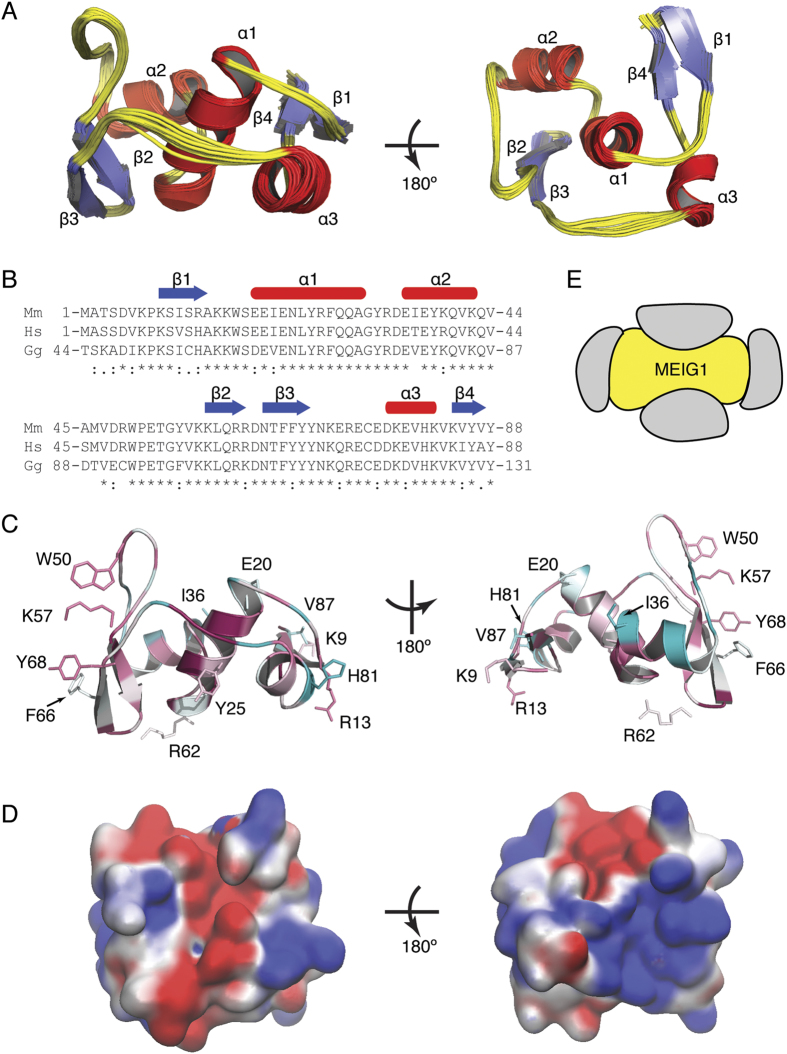
Solution structure of MEIG1. The structure of MEIG1 was determined by NMR spectroscopy. (**A**) An overlay of the 20 lowest energy structures (ordered residues 8–88) shows that MEIG1 adopts a unique fold. Two α-helices (α2, α3 – shown in red) and two small β-sheets (β1/β4, β2/β3 – shown in blue) surround a central alpha helix (α1) to form relatively flat dumbbell shaped molecule. (**B**) A clustal omega alignment^42^ of the mouse (Mm), human (Hs), chicken (Gg) MEIG1 primary sequence is shown with secondary structure elements (blue arrow – sheet, red oval – helix) indicated above the sequence. (**C**) A schematic diagram shows that the overall shape of MEIG1 (yellow) provides four potential binding surfaces to interact with associated proteins (grey). (**D**) A ribbon diagram depicting MEIG1 in two different orientations (rotated by 180°) is colored based on conservation score as determined by ConSurf^20^. The colors range from turquoise (most variable) to maroon (most conserved). The 12 amino acids identified for mutagenesis are depicted as sticks and labeled. (**E**) The solvent accessible surface of MEIG1in two different orientations (rotated by 180°) is colored according to electrostatic charge as determined by APBS and PDB2PQR^23^,^24^,^25^.

The MEIG1 protein is well-behaved in isolation and remains a monomer at high concentration based on both the elution profile from size exclusion chromatography as well as the narrow linewidths observed in the 2D ^15^N-HSQC spectrum (Fig. 1). The solution structure of MEIG1 reveals a unique fold that provides a large surface area for binding to other proteins. The structure was well determined experimentally (Table 1) with an RMSD of 0.4 Å for backbone and 1.0 Å for all heavy atoms of ordered residues (amino acids 8–88). An overlay of the twenty lowest energy structures (Fig. 2A) shows a very tight ensemble of structures with only small variations in the backbone. The protein adopts a very unique fold without any close structural homologues identified by either VAST or DALI searches^17^,^18^. The first alpha helix (α1) functions as a central axial hub. Two additional helices (α2, α3) and a small beta sheet (β1/β4) and a beta hairpin (β2/β3) pack circumferentially around α1. This organization leads to an irregular disc shaped structure that appears as a lopsided dumbbell when viewed on edge (Fig. 2A,B). The smaller end of the dumbbell collapses around a core formed largely by branch chained hydrophobic residues while the larger end of the dumbbell collapses around a core of aromatic side chains. In this manner, the hydrophobic core of the protein is distributed concentrically around the central α-helix. Previous studies have suggested MEIG1 can form a homodimer through intermolecular disulfide bond formation^5^. However, the single cysteine residue in MEIG1 is completely buried with a solvent exposed surface area of less than 1%. Therefore, the conformation of the protein precludes intermolecular disulfide bond formation in the absence of large structural rearrangements or unfolding.

Given this unique fold, we investigated whether evolutionary conservation is consistent with the formation of a relatively distributed hydrophobic core. Sequence conservation analysis was performed with the online ConSurf Server (http://consurf.tau.ac.il/)^19^,^20^ which identifies homologous proteins, performs a multiple sequence alignment, and calculates position specific conservation scores using an empirical Bayesian algorithm. The results are shown in Fig. 2C in which each residue of MEIG1 is colored according to conservation score. The central helix and core residues show the highest conservation while solvent exposed and disordered residues show the least. The second α-helix and subsequent loop region shows the lowest conservation for the structured portion of the protein. This pattern of conservation is consistent with the central helix forming the central core of the protein structure.

The overall fold and dumbbell shape of the protein provides a fairly large surface area for binding to other proteins. The solvent accessible surface area for the structured domain (amino acids 8–88) is 6014.5 Å^2^ (as calculated by VMD^21^, solvent radius = 1.4 Å), whereas the expected solvent accessible surface area for a globular domain comprised of 81 amino acids is 5684.6 Å^2^,^22^, for a difference of 320 Å^2^. The surface charge calculated by the APBS program^23^,^24^,^25^ shows distinct patches of positive and negative charges on opposing surfaces of the protein (Fig. 2D). In addition, three aromatic residues, Phe^66^, Tyr^68^, Trp^50^, as well as the positively charged Lys^57^ are well conserved and exposed on the large end of the dumbbell (Fig. 2C). Therefore MEIG1 presents a fairly large surface area such that associated proteins can bind charged or hydrophobic patches on either of two opposing concave surfaces or the two convex ends of the dumbbell (Fig. 2E). Hence the structure of MEIG1 is consistent with the role of the protein as an adaptor that recruits cargo to the manchette for delivery out the sperm tail.

### A solvent exposed hydrophobic patch is critical for binding PACRG

In previous studies, we have shown that MEIG1 forms a complex with PACRG within the manchette of spermatids and is necessary for the delivery of cargo and formation of the sperm tail^26^. Based on the solution structure, 12 solvent exposed, hydrophobic or charged residues were identified as potential mediators of protein-protein interactions. These 12 amino acids were mutated as described in supplemental Table 1, and binding strength between PACRG with wild-type and mutated MEIG1 proteins was tested by direct yeast two hybrid experiments.

The mutations were introduced using MEIG1/pGBKT7 as template. To test if the mutations affect MEIG1 protein expression, the wild-type and all the mutated plasmids were transformed into yeast strain AH109, and yeast lysates were prepared for Western blot analysis using an anti-MEIG1 antibody. There was no difference in MEIG1 expression level among all the mutants (Supplemental Fig. 1). The wild-type and mutated plasmids were co-transformed into AH109 together with PACRG/pGADT7, and an empty pGADT7 was also co-transformed as negative controls, and selection medium was used for yeast growth. All the yeast grew when the yeast expressed both PACRG and MEIG1, either wild-type or mutated. However, no yeast grew when empty vector was used (Supplemental Fig. 2). The results indicate that PACRG still interacts with MEIG1 even if it is mutated.

**Figure 3:**
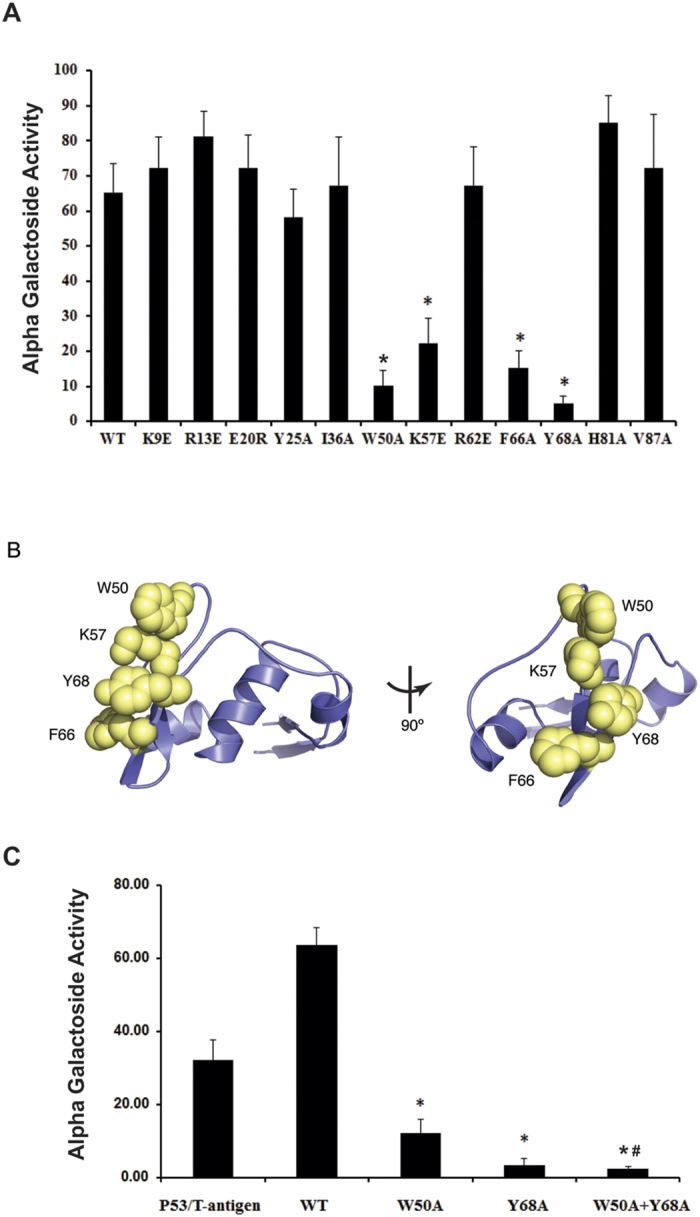
Binding strength between PACRG with wild-type and mutant MEIG1. Yeast strain AH109 were co-transformed with indicated plasmids, and binding strength was analyzed by measuring α-galactosidase activity. (**A**) Binding strength between PACRG with wild-type MEIG1 and individual MEIG1 mutants. Notice that compared to the binding strength between PACRG with wild-type MEIG1, the binding strengths in W50A, K57E, F66A, particularly Y68A, were significantly reduced. *p < 0.05. (**B**) Residues involved in binding to PACRG. The four residues critical for binding PACRG (W50, K57, F66, and Y68) are shown as yellow spheres on a blue ribbon diagram. These four residues form a contiguous surface on the large globular end of the protein. (**C**) Binding strength in double mutations. Notice that binding strength in W50A/Y68A double mutations is significantly lower than single W50A or Y68A mutation. The binding strength between PACRG and wild-type MEIG1 is even higher than the positive P53 and large T antigen. *p < 0.05 compared to wild type, and #p < 0.05 compared to Y68A mutation.

Binding strength between PACRG with wild-type MEIG1 and the mutated MEIG1 proteins was evaluated by measuring α-galactosidase activity. Of the 12 mutations, four (W50A, K57E, and F66A, particularly Y68A) significantly reduced binding (Fig. 3A). These four amino acids are solvent exposed on the convex surface of the large end of the dumbbell (Fig. 3B). These results indicate that this exposed hydrophobic patch forms the interaction interface with PACRG. To confirm that we have not disrupted the global fold of the protein, the MEIG1^Y68A^ mutant was isotopically labeled and a 2D ^15^N-HSQC compared with that of wild-type protein. As can be seen in Supplemental Fig. 3A,B, mutating this residue leads to relatively minor chemical shift changes for only a few resonances in the vicinity of Y68. The 2D spectrum otherwise remains quite dispersed and very similar to wild-type protein. Since chemical shifts are highly sensitive to changes in local environment, these findings confirm that MEIG1^Y68A^ maintains a native fold. In addition, each of the mutant proteins that affect binding to PACRG was analyzed by size exclusion chromatography (Supplemental Fig. 3C).The elution profiles were similar to wild type protein and appropriate for a monomeric globular domain. This study further confirms that these mutations do not unfold the protein or lead to large structural changes.

We further compared binding strength between PACRG with MEIG1 carrying single mutations (W50A and Y68A) and W50A/Y68A double mutations, and discovered that yeast co-transformed with PACRG and MEIG1 with W50A/Y68A double mutations still grew on the selection plate (Supplemental Fig. 4), however, the binding strength was significantly reduced in the double mutations (Fig. 3C).

Human PACRG is degraded, in part, by the ubiquitin-proteasome system^27^,^28^. Mouse PACRG expression was significantly increased by the proteasome inhibitor MG132 in transfected COS-1 cells, indicating that mouse PACRG is also regulated, in part, destroyed by the same mechanism^26^. As in cultured mammalian cells, mouse PACRG protein is not stable in bacteria. However, in transfected mammalian cells, PACRG protein is easily detected by the Pico system (Pierce) in Western blot analysis, but not in bacteria. The His-tag labeled PACRG protein from bacteria transformed with PACRG/pET28A plasmid was not visualized by Coomassie blue staining after IPTG induction. After standard His-tag protein purification procedure, no PACRG protein was visualized by either Coomassie blue staining or Western blot using the Pico (Pierce) system. However, a small amount of PACRG protein was indeed expressed, because it was detectable when a higher sensitivity Femto system (Pierce) was used for Western blot analysis (Supplemental Fig. 5). We concluded that in bacteria, PACRG protein is degraded quickly, and is not as stable as in the mammalian cells.

**Figure 4:**
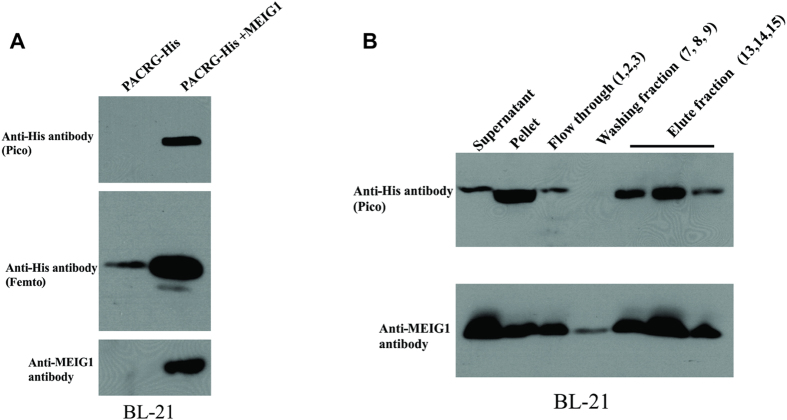
Association between MEIG1 and PACRG in bacteria. (**A**) MEIG1 stabilizes PACRG in bacteria. Notice that PACRG was only detectable by the high sensitivity Femto system in Western blot analysis when the bacteria were transformed with PACRG/pCDFDuet-1 plasmid; However, when the bacteria were transformed with PACRG/MEIG1/pCDFDuet-1 plasmid to express MEIG1 protein, PACRG was detectable by less sensitive Pico system. (**B**) PACRG and MEIG1 were co-purified in gel filtration experiments. Lysates from bacteria expressing both PACRG and MEIG1 were filtrated and Western blotting was conducted. PACRG and MEIG1 were present in the same fractions.

In cultured mammalian cells, the PACRG expression level was increased by co-expressing MEIG1, suggesting that PACRG is stabilized by MEIG1^26^. We next decided to co-express MEIG1 and PACRG in bacteria and determine if MEIG1 also protects PACRG in the prokaryotic system. Full-length mouse *Pacrg* cDNA was cloned into the upstream multiple clone site of the dual expression vector pCDFDuet-1 to create the PACRG/pCDFDuet-1 plasmid, and the translated protein was tagged with hexahistidine. The full-length mouse *Meig1* cDNA was inserted into the downstream multiple cloning site to create PACRG/MEIG1/pCDFDuet-1 plasmid. Similar to what was observed with the PACRG/pET28A expression vector, no PACRG protein was detectable when the less sensitive Pico system was used for Western blot analysis if the bacteria were transformed with PACRG/pCDFDuet-1 plasmid and induced by IPTG, but the PACRG protein was detectable when the high sensitive Femto system was used for Western blot analysis. However, when the bacteria were transformed by PACRG/MEIG1/pCDFDuet-1 plasmid, the PACRG was easily detectable with the Pico system (Fig. 4A), even though the protein was still not visualized by Coomassie blue staining. This suggests that wild-type MEIG1 also stabilizes PACRG in bacteria. Association between PACRG and MEIG1 in bacteria was further supported by the fact that the two proteins eluted in the same fractions when gel filtration experiments were conducted using the bacteria lysates. Expressing both PACRG (with His tag) and MEIG1 in bacteria yielded a small amount of soluble protein purified by Ni and gel filtration chromatography. The eluted protein was concentrated and passed over the gel filtration column a second time. Western blot analysis of the eluted protein confirms that both MEIG1 and PACRG were co-purified which confirms formation of a stable complex (Fig. 4B).

**Figure 5:**
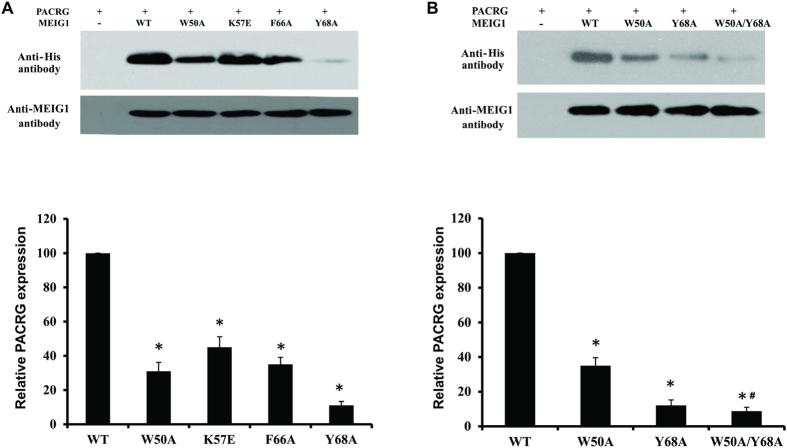
MEIG1 stabilizes PACRG in bacteria, but the ability was reduced when mutated. (**A**) The ability to stabilize PACRG is reduced in the four MEIG1 mutations. Upper panel shows a representative Western blot result from bacteria lysates. Notice that PACRG expression levels were reduced when bacteria expressed the four mutated MEIG1 proteins. Lower panel shows the relative PACRG expression levels normalized by MEIG1 protein expression levels in the four mutants compared to the wild-type MEIG1 protein. *p < 0.05 compared to wild type. (**B**) Double MEIG1 mutations cause a greater reduction in PACRG expression. Upper panel shows a representative Western blot result with indicated single and double mutations. Lower panel shows the relative PACRG expression levels normalized by MEIG1 protein expression levels. Notice that in the double mutations, PACRG level is lower than the single mutation alone. *p < 0.05 compared to wild type, and #p < 0.05 compared to Y68A mutation.

Using these systems, we tested whether the four mutants disrupted the ability of MEIG1 to stabilize PACRG in bacteria. We co-expressed the four mutant MEIG1 proteins with PACRG as described previously. Both Coomassie blue staining and Western blot analysis using the bacteria lysates showed that MEIG1 expression levels were not changed when the amino acids were mutated (Fig. 5A and Supplemental Figure 6A). However, compared to the wild-type MEIG1, PACRG expression levels were dramatically reduced in the four MEIG1 mutants, particularly for the Y68A mutation (Fig. 5A) which also showed the lowest the binding strength. We further examined the affect of the W50A/Y68A double mutation on stabilization of PACRG. Like in the single mutations, this double mutation did not change MEIG1 expression level as indicated by both Coomassie blue staining and Western blot analysis (Fig. 5B and Supplemental Figure 6B). Compared to W50A and Y68A single mutations, the PACRG expression level was even lower when co-expressed with the W50A/Y68A MEIG1 double mutation (Fig. 5B).

Hence, the four mutations we identified not only reduce binding strength but also reduce the ability of MEIG1 to stabilize PACRG in bacteria. In addition, the W50A/Y68A double mutation reduces binding strength and the ability to stabilize PACRG to a greater extent than either single mutation alone. Together, these studies establish the structure of MEIG1 and the amino acids that mediate interactions with PACRG, and open the possibility of exploring the structural mechanism for MEIG1 interaction with other proteins.

## Methods

### MEIG1 Protein expression and purification

We previously constructed the full-length mouse MEIG1/pET28A plasmid with hexahistidine tags in both N and C-termini of MEIG1 protein^8^. A stop codon was inserted after the coding region, creating the construct with only the N-terminal hexahistidine tag and intervening thrombin cleavage site. Uniform ^15^N, ^13^C labeled protein was expressed by growth in labeled M9 media^29^ and induction with 1 mM isopropyl β-D-1-thiogalactopyranoside (IPTG) at 37 °C for 4 hours. For the majority of NMR samples (including assignment and NOE data), bacteria were lysed with B-PER™ Bacterial Protein Extraction Reagent (Thermo Scientific) and the soluble fraction purified over a nickel column. To improve the yield of protein for residual dipolar coupling measurements, the bacteria were lysed in 6 M guanidine hydrochloride, 20 mM Tris pH 8.0, and the protein refolded by dialysis against 20 M Tris pH 8.0, 150 mM NaCl, 2 mM β-mercaptoethanol. The refolded protein was purified over a nickel column, the thioredoxin and hexahistidine tags removed by thrombin cleavage, and the protein isolated by gel filtration and ion exchange chromatography. The refolded protein shows nearly identical elution profile and NMR spectra as compared to protein purified under native conditions.

### NMR spectroscopy

Purified protein was buffer exchanged into 10 mM NaPO_4_, pH 6.5, 1 mM dithiothreitol, 10% ^2^H_2_O, and 0.02% sodium azide and concentrated to 0.5–1.0 mM. Standard triple resonance NMR spectra for resonance assignments, distance restraints, residual dipolar couplings, and torsion angle restraints (three dimensional HNCO, HNCACB, CCH-TOCSY, HBHA(CBCACO)NH, H(CCO)NH, C(CCO)NH,^13^C-NOESY, ^15^N-NOESY, IPAP-HNCO experiments and two dimensional IPAP-HSQC and quantitative J correlation spectroscopy experiments)^30^,^31^,^32^ were collected on Bruker Avance 700 MHz and 850 MHz instruments. Data were processed with NMRPipe^33^ and analyzed with CcpNmr^34^,^35^. Residual dipolar couplings were measured for a partially aligned sample in 5% PEG:hexanol (C_12_E_5_; r = 0.85)^36^.

### Structure calculation

Backbone torsion angle restraints were derived from chemical shift index using the TALOS-N software^37^. Hydrogen bond distance and angular restraints were introduced based on TALOS-N predictions and characteristic NOE patterns. The initial structure was calculated with automatic NOE assignments based on NOE, hydrogen bond, and torsion angle restraints using the CYANA program^38^,^39^. The final structure was refined based on NOE distance, hydrogen bond distance and angle, torsion angle, and residual dipolar coupling restraints as well as a torsion angle database potential of mean force^40^ using the Xplor-NIH software package^41^.

### Other plasmid constructs

MEIG1/pGBKT7 was constructed previously^8^. Full-length mouse *Pacrg* coding region was amplified by PCR and cloned into pGADT7 vector to create PACRG/pGADT7 plasmid. Mouse full-length *Pacrg* cDNA was also cloned into pET28A or pCDFDuet-1 vectors in order to express PACRG in bacteria. Based on the PACRG/pCDFDuet-1 plasmid, full-length mouse *Meig1* cDNA was inserted into the second multiple cloning site of the pCDFDuet-1 vector to create PACRG-MEIG1-pCDFDuet-1 plasmid. All related primers are listed in the supplemental table.

### Site-directed mutagenesis

The MEIG1/pGBKT7, PACRG-MEIG1-pCDFDuet-1 or MEIG1/pET28A plasmids were used as templates, and the indicated amino acids of MEIG1 protein were mutated using a QuikChange XL Site-directed Mutagenesis kit (Agilent Technologies, Santa Clara, CA) and the mutations were confirmed by DNA sequencing. All related primers are listed in the supplemental table.

### Direct yeast two-hybrid experiment

To detect interaction between wild-type mouse PACRG protein and wild-type mouse MEIG1 or mutant MEIG1 proteins, PACRG/pGADT7 and wild-type or mutant MEIG1/pGBKT7 were co-transformed into the AH109 host strain using the Match-Maker two-hybrid System 3 (Clontech). Expression of both proteins (PACRG and MEIG1) was analyzed by Western blot. The AH109 transformants harboring both MEIG1/pGBKT7 (wild-type or mutant) and PACRG/pGADT7 were streaked out in complete drop-out medium (SCM) lacking tryptophan, leucine and histidine to test for histidine prototrophy. Two plasmids containing simian virus (SV) 40 large T antigen (LgT) in pGADT7 and p53 in pGBKT7 were co-transformed into AH109 and used as positive controls. His3 is a reporter gene in the system.

### Gel filtration experiments

The dual expression plasmid was transformed into Rosetta II (DE3) (Invitrogen) *E. coli* strain, grown in Luria Bertani medium, and induced with 1 mM isopropyl-β-d-thiogalactopyranoside at an A_600_ ~ 0.8 for 2 hours. The bacterial pellets from 1L of growth media were resuspended in 30 mL of B-PER reagent (Thermo Scientific) and expressed protein purified from the lysis supernatant by Nickel affinity and gel filtration (Superdex 75 26/60, GE Healthcare) chromatography. The purified protein was concentrated and passed over the gel filtration column a second time before Western blot analysis.

### Analysis of α-galactosidase reporter gene activity

α-Galactosidase reporter gene activity was measured following instructions provided by the manufacturer (Match-Maker two-hybrid System 3; Clontech). Briefly, AH109 clones co-transformed with indicated pairs of PACRG/pGADT7 and wild-type or mutant MEIG1/pGBKT7 were cultured in SCM-Leu-Trp medium to optical density (OD)_600_ nm –1.0. One millilitre of culture from each inoculation (in triplicate) was centrifuged in a 1.5 ml centrifuge tube at 10,000 ×g for 2 min, at 22 °C. The supernatants, which contained α-Galactosidase, were carefully transferred to fresh tubes on ice. Aliquots of 120 μl of culture supernatant were mixed with 360 μl of assay buffer [prepared by mixing two volumes of 0.5 mol/L sodium acetate pH 4.5 and 1 vol of 100 mmol/L of p-nitrophenyl α-D-galactopyranoside (PNP-α-Gal)]. The reaction mixes were incubated at 30 °C for 3 h before stopping the reaction with the addition of 520 μl of stop buffer (1 mol/L sodium carbonate). The OD_410_ of each reaction was recorded by a spectrophotometer using a reaction mix containing SCM-Leu-Trp medium in the place of culture supernatant as the blank. The activity was expressed either as arbitrary units or percentage of wild type gene (as a way to record interaction strength between PACRG and MEIG1 mutants). Each assay was repeated five times and the average was reported.

### Western blot analysis

Indicated bacteria or yeast protein lysates were heated to 95 °C for 10 min in 4× sample buffer, loaded onto 10% or 15% SDS-polyacrylamide gels, electrophoretically separated, and transferred to polyvinylidene difluoride membranes. The membranes were blocked and then incubated with antibodies against PACRG or MEIG1 overnight at 4 °C. Both antibodies were generated by our laboratory previously^16^. After washing, the blots were incubated with an anti-mouse or anti-rabbit immunoglobulin conjugated to horseradish peroxidase for 1 h at room temperature. PACRG or MEIG1 proteins were detected with the Super Signal Pico Chemiluminescent or Femto Maximum Sensitive system (Pierce). To compare expression level of target proteins, the films were scanned to JPEG files, and Images J software was used to calculate relative expression levels.

### Statistical analysis

ANOVA test was used to determine statistical differences, the 2-tailed student’s *t*-test was used for comparison of frequencies. Significance is defined as *P* < 0.05.

### Accession Numbers

The coordinates and NMR restraints for MEIG1 have been deposited in the Protein Data Bank (PDB ID: 2n2y); the NMR assignments have been deposited in the Biological Magnetic Resonance Bank (BMRB accession: 25627).

## Additional Information

**How to cite this article**: Li, W. *et al.* Dissecting the structural basis of MEIG1 interaction with PACRG. *Sci. Rep.* **6**, 18278; doi: 10.1038/srep18278 (2016).

## Supplementary Material

Supplementary Information

## Figures and Tables

**Table 1:** NMR and refinement statistics.

NMR distance and dihedral constraints	
Distance constraints	
Total NOE	1234
Intra-residue	368
Inter-residue	
Sequential (|*i* – *j*| = 1)	323
Medium-range (|*i* – *j*| ≤ 4)	187
Long-range (|*i* – *j*| > 5)	356
Hydrogen bonds	38
Total dihedral angle restraints	172
ϕ	78
ψ	76
χ1	18
Total RDCs	
NH	60
NC’	44
Q%	
NH	1.8
NC’	32.1
**Structure statistics**	
Violations (mean and s.d.)	
Distance constraints (Å)	0.002 ± 0.001
Dihedral angle constraints (°)	0.9 ± 0.4
Max. dihedral angle violation (°)	6.8
Max. distance constraint violation (Å)	0.07
Deviations from idealized geometry	
Bond lengths (Å)	0.0031 ± 0.0002
Bond angles (°)	0.45 ± 0.03
Impropers (°)	0.40 ± 0.03
Average pairwise r.m.s. deviation** (Å)	
Heavy	1.0 ± 0.1
Backbone	0.4 ± 0.1
Ramachandran plot summary	
Most favored regions	90.5%
Additionally allowed regions	9.4%
Generously allowed regions	0.1%
Disallowed regions	0.1%

^**^Pairwise r.m.s. deviation from the mean was calculated among 20 lowest energy refined structures for ordered residues.
