# Supplementary material for: Dissecting the structural basis of MEIG1 interaction with PACRG
Source: Sci Rep. 2016 Jan 4;6:18278. doi: 10.1038/srep18278 (PMC4698733; doi:10.1038/srep18278)
Supplement: Supplementary Information [file srep18278-s1.pdf]

Dissecting the structural basis of MEIG1 interaction with PACRG

Wei Li[1*], Ninad M Walavalkar[2*], William A Buchwald[2], Maria E Teves[1], Ling Zhang[1, 3], Hong Liu[1, 3], Stephanie Bilinovich [2], Darrell L Peterson[4], Jerome F Strauss III [1,4], David C Williams Jr[2#], Zhibing Zhang[1, 4#]

1 Department of Obstetrics and Gynecology, Virginia Commonwealth University, Richmond, VA, 23298. 2. Department of Pathology and Laboratory Medicine, University of North Carolina, Chapel Hill, NC 27599. 3 School of Public Health, Wuhan University of Science and Technology, Wuhan, Hubei, 430065, China.4Department of Biochemistry & Molecular Biology, Virginia Commonwealth University, Richmond, VA, 23298.

* These authors contributed equally in this study.

# Address correspondence to: David C. Williams Jr,MD, PhD
Associate Professor
Department of Pathology and Laboratory Medicine
University of North Carolina
Chapel Hill, NC 27599
Tel: 919-843-9949
Email:david_willjr@med.unc.edu

Or Zhibing Zhang, MD, PhD
Associate Professor
Department of Obstetrics/Gynecology
Virginia Commonwealth University
1101 E Marshall Street
Richmond, VA, 23298
Email: zhibing.zhang@vcuhealth.org

Supplemental figure legends

Supplemental Figure 1. MEIG1 protein expression level was not changed by mutations in yeast.

Protein lysates were prepared from the yeast expressing wild-type and the twelve mutated MEIG1, and separated by SDS-PAGE gels for Western blotting analysis to examine MEIG1 protein expression (A) to Coomassive blue staining (B). Three independent experiments were conducted, and statistic difference in MEIG1 expression was analyzed between wild-type and mutant MEIG1 proteins. There was no significant difference in MEIG1 protein expression level (C).

Supplemental Figure 2. Examination of interaction of PACRG with wild-type or single amino acid mutation of MEIG1 by direct yeast two-hybrid experiments.

Pairs of PACRG and wild-type or mutated MEIG1 plasmids were co-transformed into AH109 yeast, and the transformed yeast were grew on selection plates (lacking tryptophan, leucine and histidine) (A) or non-selection plates (lacking tryptophan and leucine) (B). Pair of p53 and large T antigen was used as a positive control, and pair of empty vectors was used as a negative control. Notice that all the yeast grew on the non-selection plate (B). Yeast expressing two proteins (p53/large T antigen or PACRG/wild-type or mutated MEIG1) grew on selection plate, but not the negative control (A).

Supplemental Figure 3. The mutations do not disrupt the structure of MEIG1.

(A) An overlay of wild-type (red) and Y68A mutant (blue) MEIG1 2D ^{15}N-HSQC spectra show that both spectra are very similar with only relatively small scattered chemical shift changes. Peaks that shift significantly are labeled with their assignments. (B) A ribbon diagram of MEIG1 depicts with sticks the mutated residue (Y68) colored red, residues that show chemical shift changes colored yellow, and the two showing largest chemical shift changes (K58 and F66) in orange. (C) Wild type MEIG1 and all mutants that disrupt binding to PACRG (W50A, K57E, F66A, Y68A, and W50A/Y68A) were purified over a nickel column and analyzed by size exclusion chromatography on a Superdex 75 10/300 (GE Healthcare). The elution profile shows that these mutants behave similarly to wild type and confirm that mutation has not caused large structural changes or unfolded the protein.

Supplemental Figure 4. Examination of interaction of PACRG with MEIG1 with W50A/Y68A double mutation by direct yeast two-hybrid experiments.

Experiment procedure is the same as described in Supplemental Figure 2, except that pair of PACRG and W50A/Y68A double mutated MEIG1 plasmids were co-transformed into AH109 yeast. Notice that the yeast grew on both non-selection and selection plates.

Supplemental Figure 5. Low expression level of mouse PACRG in induced BL 21 bacteria.

Bacteria (BL21) were transformed by PACRG/PET28A plasmid and induced by 1mM IPTG, and the total bacteria lysates were separated in 12% SDS-PAGE gels. PACRG band was not visualized by either Coomassie blue staining (upper panel) or Western blot analysis using less sensitive Pico-system (middle panel). However, the PACRG was detected by more sensitive Femto system (lower panel).

Supplemental Figure 6. MEIG1 protein expression level was not changed by mutations in bacteria.

Bacteria (BL21) expressing wild-type MEIG1 and MEIG1 with single mutations (A) or W50A/Y68A double mutations (B) were collected and the total lysates were separated in 15% SDS-PAGE gels, followed by Coomassie blue staining. These mutations did not change MEIG1 expression levels.

Supplemental table 1. Proposed MEIG1 mutation in the study.

**Table 1 Proposed**	**MEIG1 mutations**
Large globular domain	W50A, K57E, F66A, Y68A
Small globular domain	K9E, R13E, H81A, V87A
Opposing clefts between globular domains	E20R, Y25A, I36A, R62E

Supplemental table 2. Sequence of oligonucleotides used in this study

Oligonucleotide names	Primer sequences
MEIG1 K9E forward	5'-cttctgacgtgaaaccagaatcaataagtcgtgcc-3'
MEIG1 K9E reverse	5'-ggcacgacttattgattctggtttcacgtcagaag-3'
MEIG1 R13E A forward	5'-ccaaaatcaataagtcgagccaagaaatggtcagagg-3'
MEIG1 R13E A reverse	5'-cctctgaccatttcttggctcgacttattgattttgg-3'
MEIG1 R13E forward	5'-ccaaaatcaataagtgaagccaagaaatggtcagagg-3'
MEIG1 R13E reverse	5'-cctctgaccatttcttggcttcacttattgattttgg-3'
MEIG1 E20Rforward	5'-cgtgccaagaaatggtcagagcgaatagaaaatctgtacagatt-3'
MEIG1 E20Rreverse	5'-aatctgtacagattttctattcgctctgaccatttcttggcacg-3'
MEIG1 Y25A forward	5'-cagaggaaatagaaaatctggccagatttcaacaagcagg-3'
MEIG1 Y25A reverse	5'-cctgcttgttgaaatctggccagattttctatttcctctg-3'
MEIG1 I36A forward	5'-caagcaggatatcgggatgaagctgaatataaacaagtgaaac-3'
MEIG1 I36A reverse	5'-gtttcacttgtttatattcagcttcatcccgatatcctgcttg-3'
MEIG1 W50A forward	5'-gttgccatggtcgaccgagcgccagagacagggtacgt-3'
MEIG1 W50Areverse	5'-acgtaccctgtctctggcgctcggtcgaccatggcaac-3'
MEIG1 K57E forward	5'-cagagacagggtacgtggagaaacttcagcggagg-3'
MEIG1 K57Ereverse	5'-cctccgctgaagtttctccacgtaccctgtctctg-3'
MEIG1 R62E forward	5'-cgtgaagaaactcagcgggaggacaatactttcttctac-3'
MEIG1 R62E reverse	5'-gtagaagaaagtattgtcctcccgctgagtttcttcacg-3'
MEIG1 F66A G forward1	5'-cggagggacaatactgtcttctactacaaca-3'
MEIG1 F66A G reverse1	5'-tgttgtagtagaagacagtattgtccctccg-3'
MEIG1 F66A forward2	5'-cggagggacaatactgccttctactacaacaa-3'
MEIG1 F66A reverse2	5'-ttgttgtagtagaaggcagtattgtccctccg-3'
MEIG1 Y68A forward	5'-gagggacaatactttcttcgcctacaacaaagagagggagtg-3'
MEIG1 Y68A reverse	5'-cactccctctctttgttgtaggcgaagaaagtattgtccctc-3'
MEIG1 H81A forward	5'-gtgcgaggacaaggaggtcgccaaagtgaaggtttacgtc-3'

MEIG1 H81A reverse	5’-gacgtaaaccttcactttggcgacctccttgtcctcgcac-3’
MEIG1 V87A forward	5’-caaagtgaaggtttacgcctactgagctgcagcg-3’
MEIG1 V87A reverse	5’-cgctgcagctcagtaggcgtaaaccttcactttg-3’
PACRGpET28A forward	5’-gctagcccagacaagatgccgaagagg-3’
PACRGpET28Areverse	5’-gaattctcagttcagcaagcacgactc-3’
MEIG1stopcodeforward	5’-gaaggtttacgtctactaagcttgcggccgcac-3’
MEIG1stpopcodereverse	5’-gtgcggccgcaagcttagtagacgtaaaccttc-3’
PACRG pGADT7forward	5’-gaattcatgccgaagaggactaaactg-3’
PACRG pGADT7reverse	5’-ggatcctcagttcagcaagcacgactc-3’
PACRG pCDduet-1forward	5’-gaattcggtgccgcgcggcagcatgccgaagaggactaaactg-3’
PACRG pCDduet-1reverse	5’-gtcgactcagttcagcaagcacgactc-3’
MEIG1 pCDduet-1forward	5’-agatctcatggctacttct6gacgtgaaac-3’
MEIG1 pCDduet-1reverse	5’-ctcgagtcagtagacgtaaaccttcac-3’

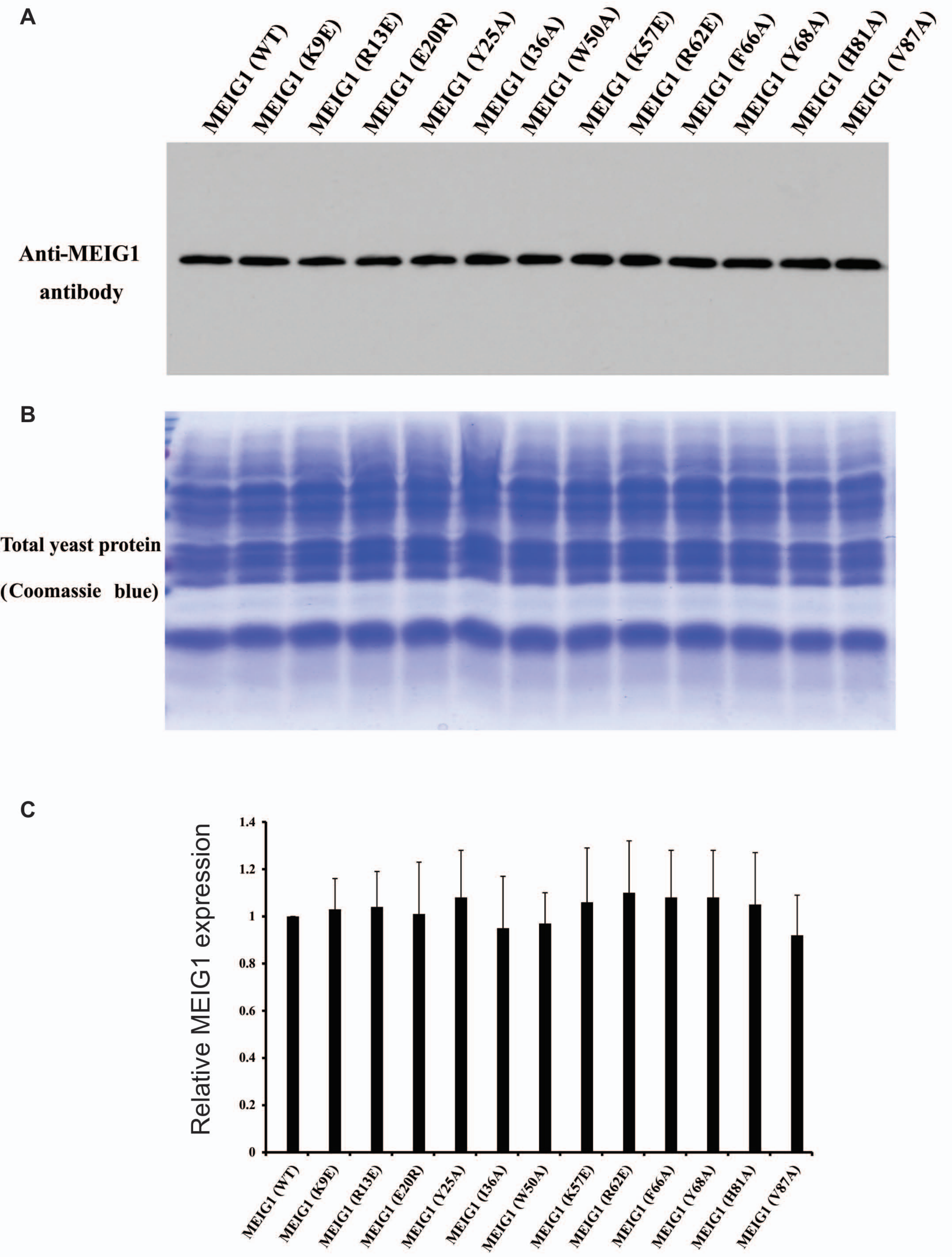
A
MEIG1 (WT)
MEIG1 (K9E)
MEIG1 (R13E)
MEIG1 (E20R)
MEIG1 (Y25A)
MEIG1 (I36A)
MEIG1 (W50A)
MEIG1 (K57E)
MEIG1 (R62E)
MEIG1 (F66A)
MEIG1 (Y68A)
MEIG1 (H81A)
MEIG1 (V87A)
Anti-MEIG1 antibody
B
Total yeast protein
(Coomassie blue)
C
Relative MEIG1 expression
1.4
1.2
1
0.8
0.6
0.4
0.2
0
MEIG1 (WT)
MEIG1 (K9E)
MEIG1 (R13E)
MEIG1 (E20R)
MEIG1 (Y25A)
MEIG1 (I36A)
MEIG1 (W50A)
MEIG1 (K57E)
MEIG1 (R62E)
MEIG1 (F66A)
MEIG1 (Y68A)
MEIG1 (H81A)
MEIG1 (V87A)

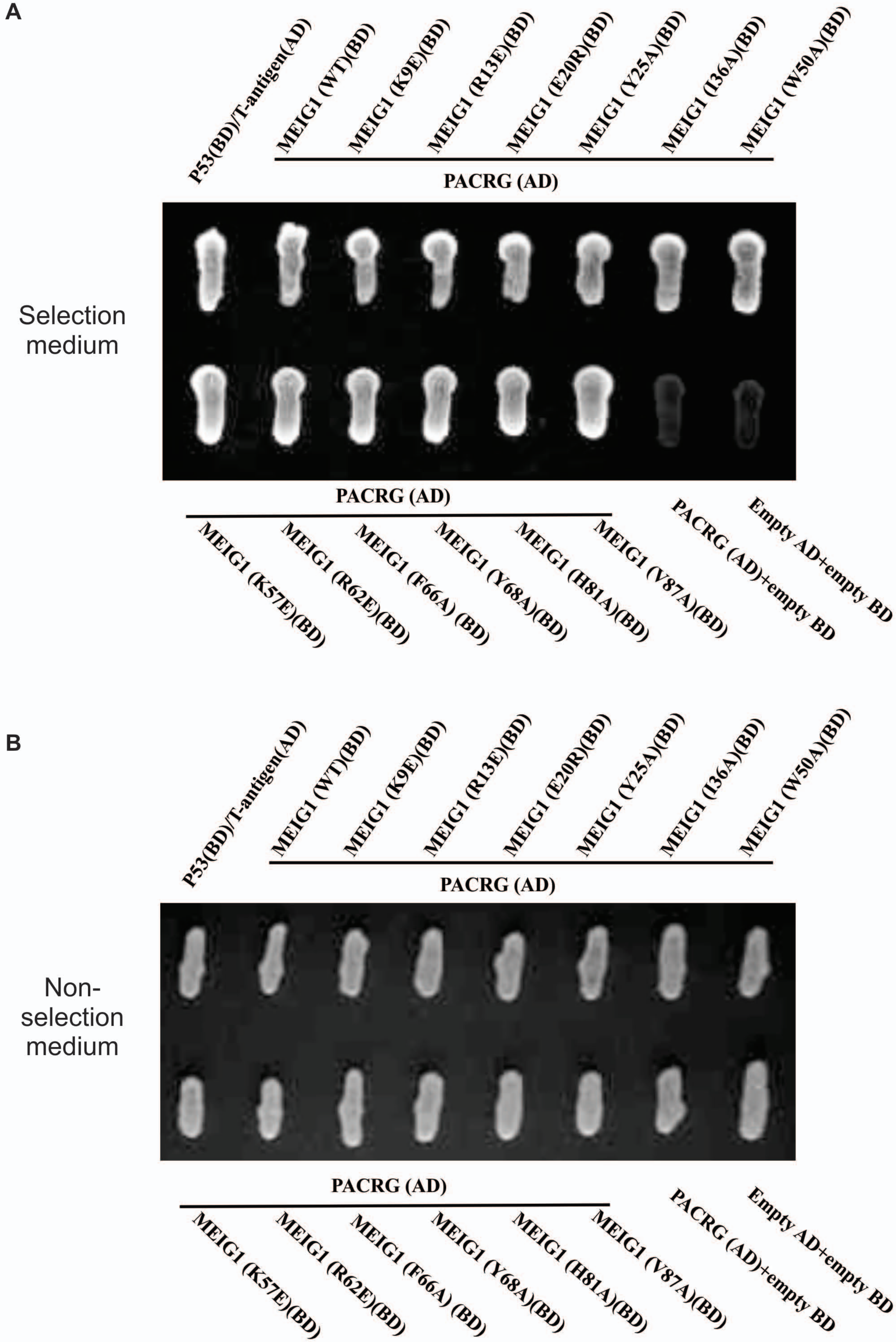

A
P53(BD)/T-antigen(AD)
MEIG1 (WT)(BD)
MEIG1 (K9E)(BD)
MEIG1 (R13E)(BD)
MEIG1 (E20R)(BD)
MEIG1 (Y25A)(BD)
MEIG1 (I36A)(BD)
MEIG1 (W50A)(BD)
PACRG (AD)
Selection medium
PACRG (AD)
MEIG1 (K57E)(BD)
MEIG1 (R62E)(BD)
MEIG1 (F66A) (BD)
MEIG1 (Y68A)(BD)
MEIG1 (H81A)(BD)
MEIG1 (V87A)(BD)
PACRG (AD)+empty BD
Empty AD+empty BD
B
P53(BD)/T-antigen(AD)
MEIG1 (WT)(BD)
MEIG1 (K9E)(BD)
MEIG1 (R13E)(BD)
MEIG1 (E20R)(BD)
MEIG1 (Y25A)(BD)
MEIG1 (I36A)(BD)
MEIG1 (W50A)(BD)
PACRG (AD)
Non-selection medium
PACRG (AD)
MEIG1 (K57E)(BD)
MEIG1 (R62E)(BD)
MEIG1 (F66A) (BD)
MEIG1 (Y68A)(BD)
MEIG1 (H81A)(BD)
MEIG1 (V87A)(BD)
PACRG (AD)+empty BD
Empty AD+empty BD

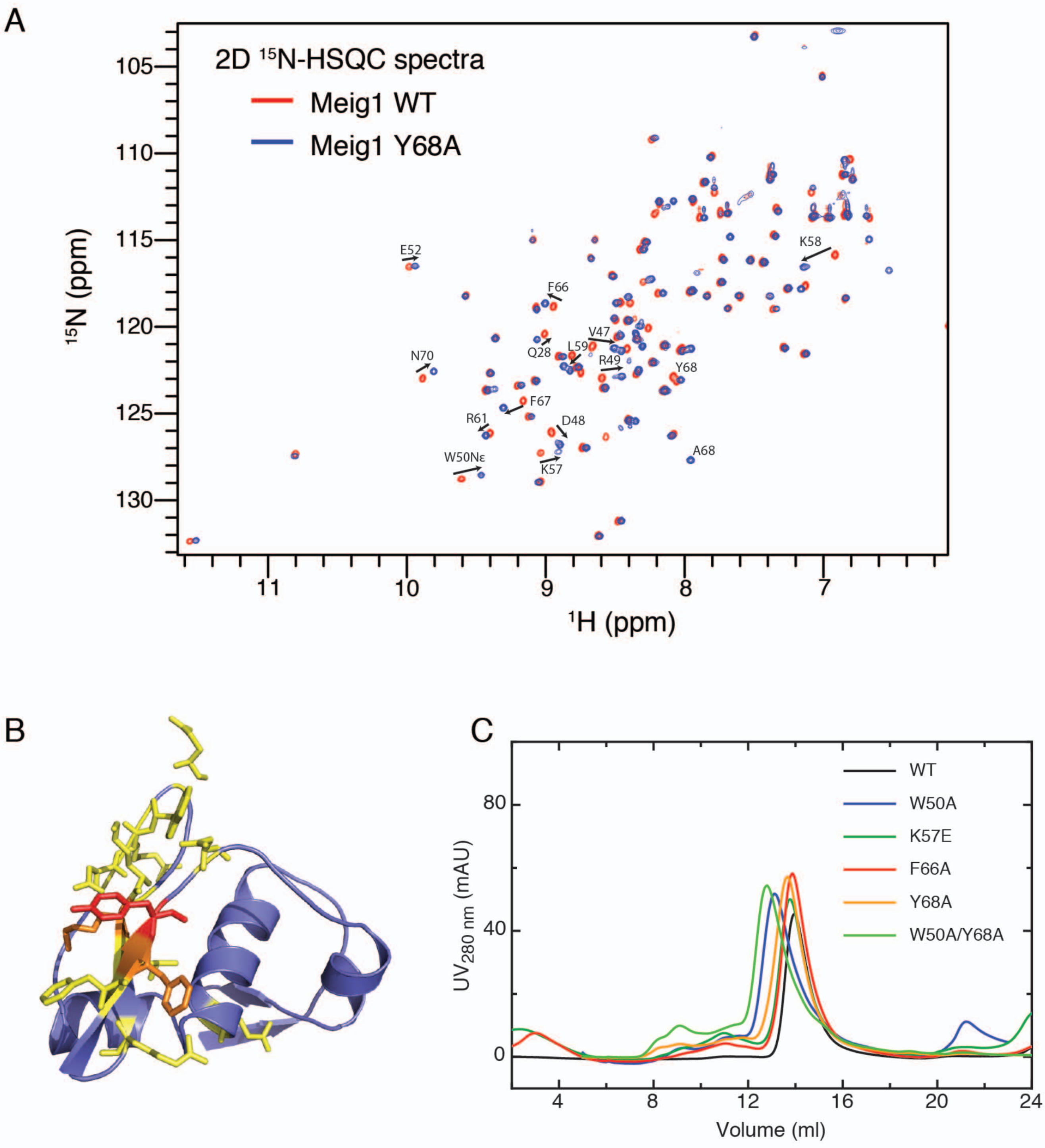
A
2D ¹⁵N-HSQC spectra
Meig1 WT
Meig1 Y68A
105
110
115
120
125
130
¹⁵N (ppm)
11
10
9
8
7
¹H (ppm)
E52
K58
F66
V47
Q28
L59
N70
R49
Y68
F67
R61
D48
W50Nε
K57
A68
B
C
WT
W50A
K57E
F66A
Y68A
W50A/Y68A
80
40
0
UV280 nm (mAU)
4
8
12
16
20
24
Volume (ml)

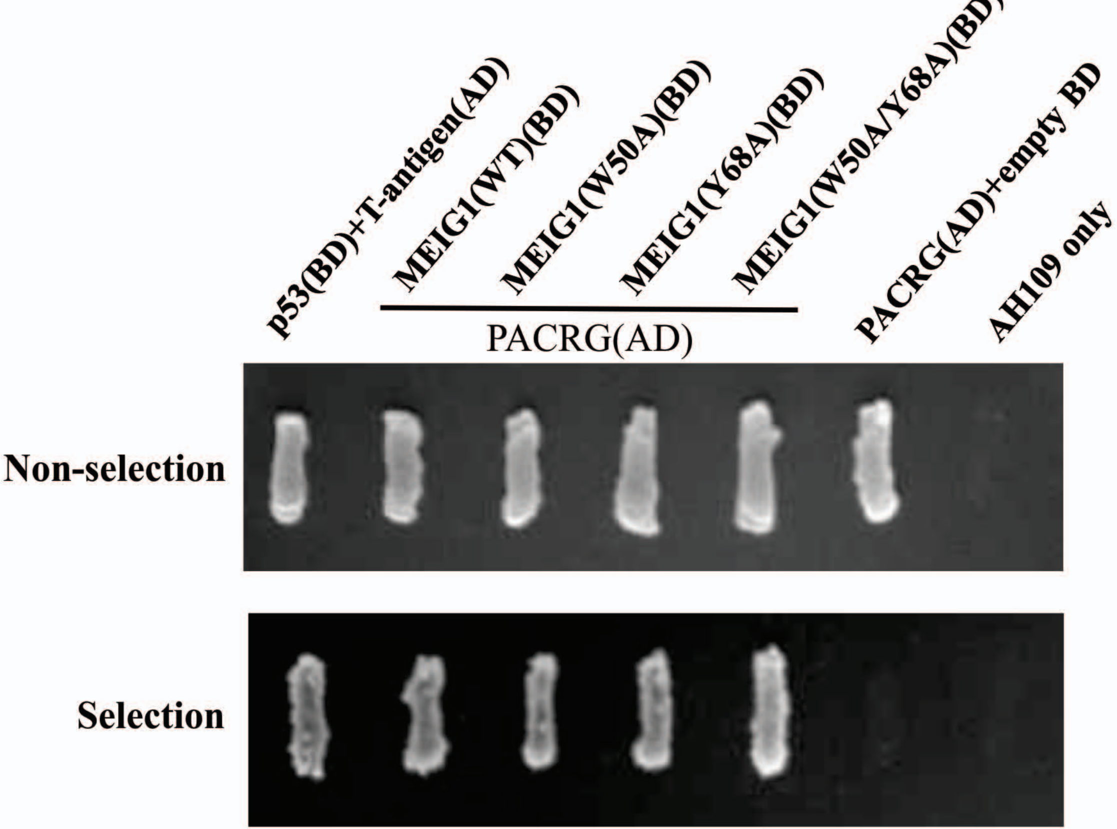
p53(BD)+T-antigen(AD)
MEIG1(WT)(BD)
MEIG1(W50A)(BD)
MEIG1(Y68A)(BD)
MEIG1(W50A/Y68A)(BD)
PACRG(AD)
PACRG(AD)+empty BD
AH109 only
Non-selection
Selection

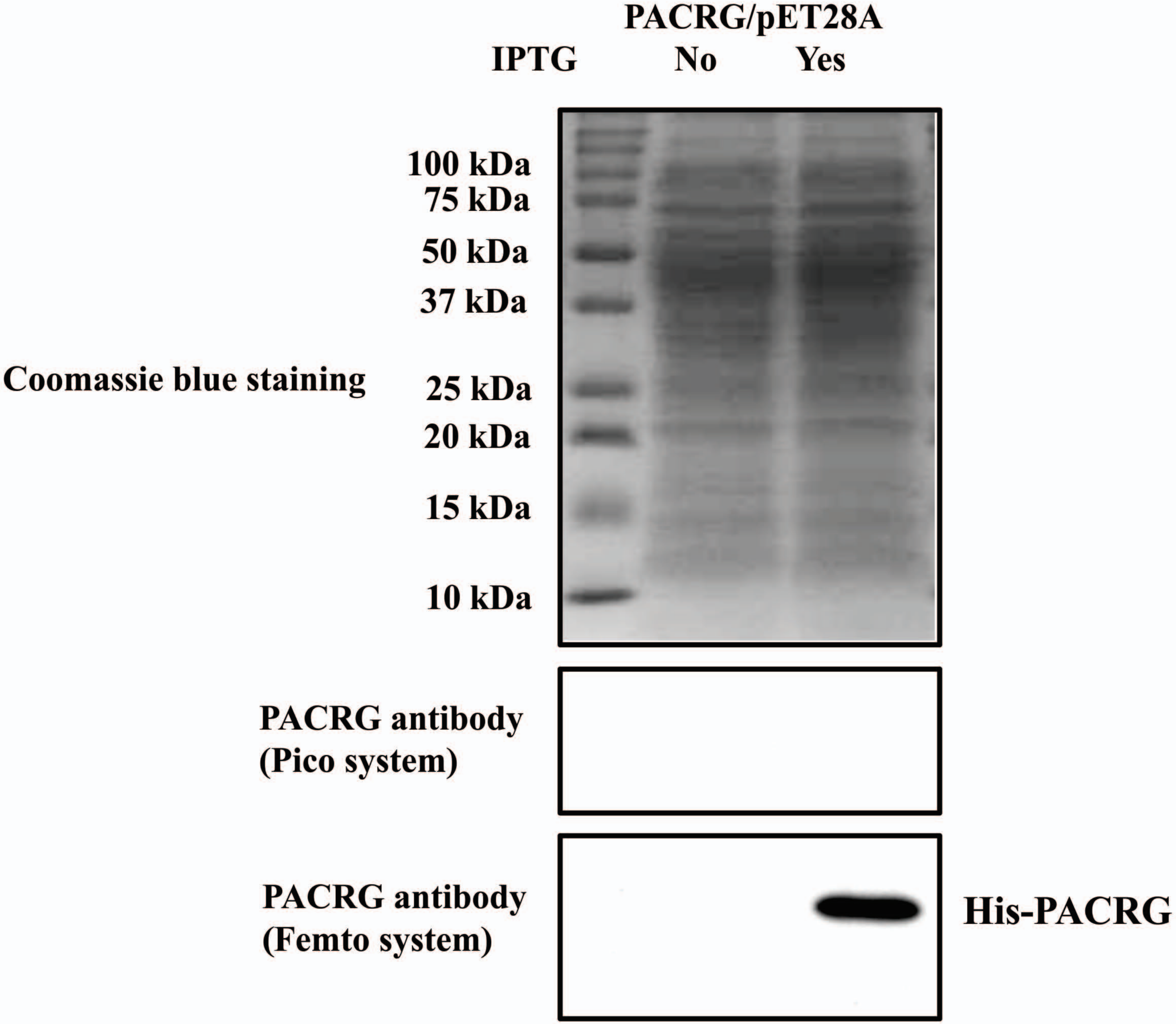
PACRG/pET28A
IPTG
No
Yes
100 kDa
75 kDa
50 kDa
37 kDa
Coomassie blue staining
25 kDa
20 kDa
15 kDa
10 kDa
PACRG antibody
(Pico system)
PACRG antibody
(Femto system)
His-PACRG

A

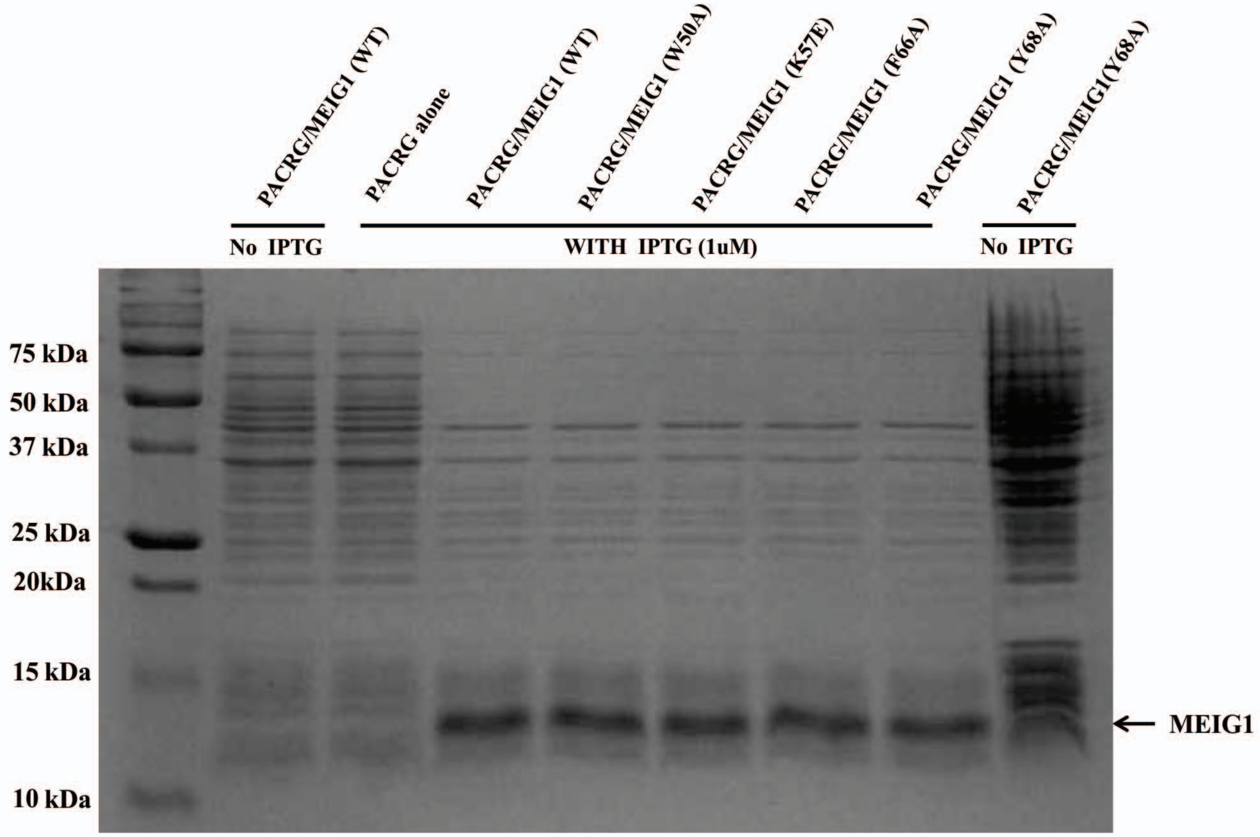

PACRG/MEIG1 (WT)
PACRG alone
PACRG/MEIG1 (WT)
PACRG/MEIG1 (W50A)
PACRG/MEIG1 (K57E)
PACRG/MEIG1 (F66A)
PACRG/MEIG1 (Y68A)
PACRG/MEIG1(Y68A)
No IPTG
WITH IPTG (1uM)
No IPTG
75 kDa
50 kDa
37 kDa
25 kDa
20kDa
15 kDa
10 kDa
MEIG1

B

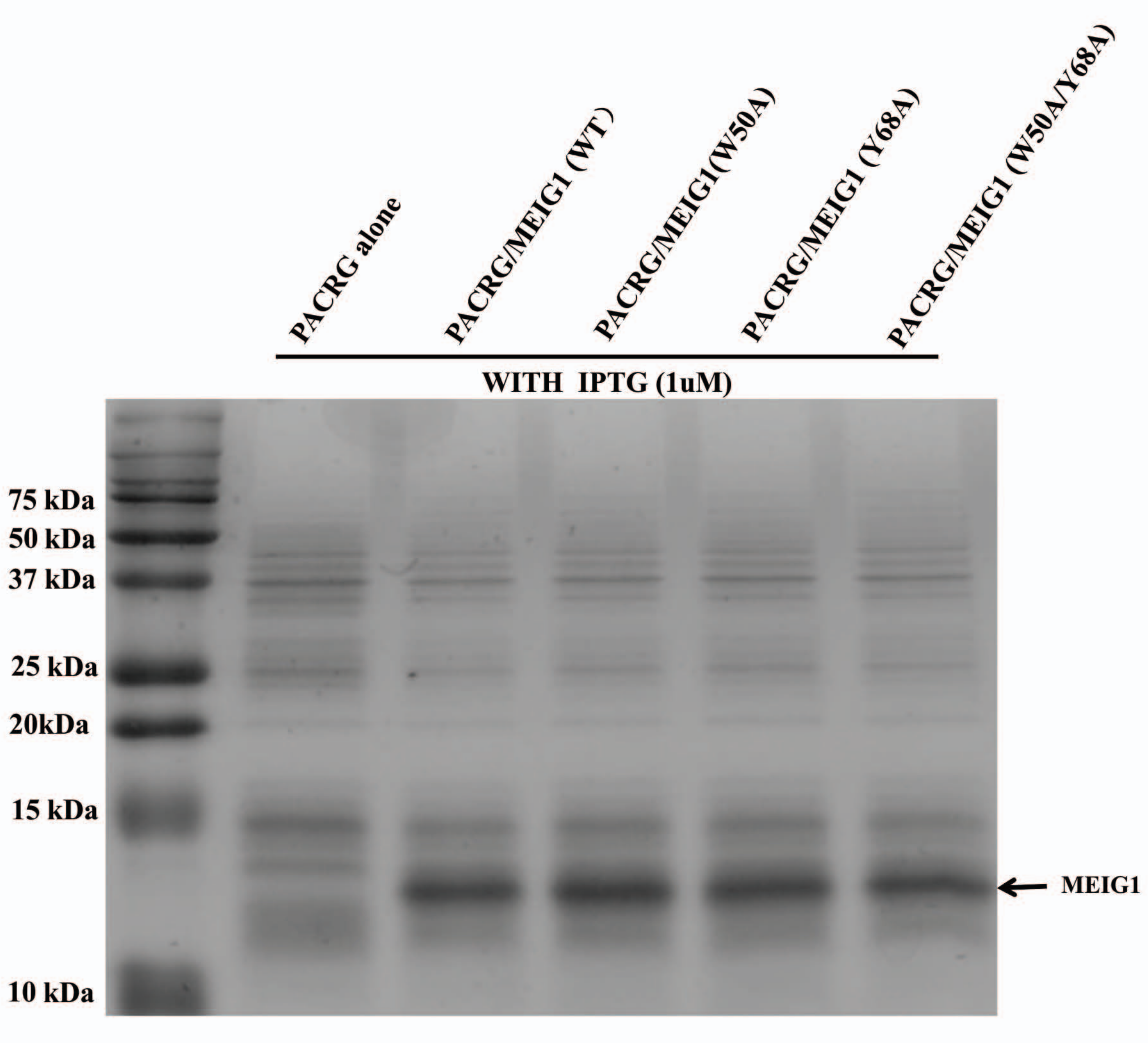

PACRG alone
PACRG/MEIG1 (WT)
PACRG/MEIG1(W50A)
PACRG/MEIG1 (Y68A)
PACRG/MEIG1 (W50A/Y68A)
WITH IPTG (1uM)
75 kDa
50 kDa
37 kDa
25 kDa
20kDa
15 kDa
10 kDa
MEIG1
